# Patients with indolent lymphomas are at high risk of infections: experience from a German outpatient clinic

**DOI:** 10.1186/s12865-022-00536-x

**Published:** 2023-01-11

**Authors:** Christoph Lutz, Stefan Feiten, Geothy Chakupurakal, Jochen Heymanns, Jörg Thomalla, Christoph van Roye, Rudolf Weide

**Affiliations:** 1 Praxis für Hämatologie und Onkologie Koblenz, Neversstr. 5, 56068 Koblenz, Germany; 2 Institut für Versorgungsforschung in der Onkologie, Koblenz, Germany

**Keywords:** Infections, Immunodeficiency, Hypogammaglobulinemia, Lymphomas, Indolent B-NHL, Longitudinal, Control group, Treatment data

## Abstract

**Background:**

Patients with indolent B-cell non-Hodgkin lymphomas (B-NHLs) have an increased risk of infections which is caused by pathomechanisms of the diseases itself but also as a result of anti-tumor therapy. Especially the effects of anti-CD20 antibodies are well understood as these lead to decreased antibody production. Most studies regarding immunodeficiency in B-NHLs were conducted with multiple myeloma and chronic lymphocytic leukemia patients. As these studies not always represent the general population we collected and analyzed real world data from patients with indolent lymphomas and a control group (CG).

**Results:**

Patients with B-NHLs undergoing therapy or who were regularly monitored in a watch and wait approach had, over the time of one year, an increased rate of infections compared to the CG of 145 healthy volunteers (mean: 11.66 vs. 7.13 infections per 1000 days). Consistent with this finding B-NHL patients received more antibiotic treatment (mean: 11.17 vs. 6.27 days) and were more often hospitalized than persons from the CG (mean: 5.19 vs. 0.99 days per 1000 days). Lymphoma patients without immunodeficiency had a lower infection rate than patients with non-symptomatic and symptomatic immunodeficiency (mean: 10.91 vs. 12.07 and 12.36 per 1000 days). The number of infections differed statistically significant for the subgroups and CG (7.13 per 1000 days). Patients with symptomatic immunodeficiency were mostly treated with regular immunoglobulin substitutions and infection rates were comparable to those of patients with asymptomatic immunodeficiency.

**Conclusions:**

Our data suggest the use of an approach with regular immune monitoring including the measurement of immunoglobulin levels and regular appointments for clinical assessment of all indolent lymphoma patients in order to identify patients with increased risk of infections. It also raises the question if patients with immunodeficiency should be treated more often with regular immunoglobulin substitution, but so far more studies are necessary to answer this question.

**Supplementary Information:**

The online version contains supplementary material available at 10.1186/s12865-022-00536-x.

## Background

Patients with hematological malignancies have an increased risk of infections [1, 2]. This susceptibility to infections is caused by malignant infiltration in hematopoietic and especially lymphopoietic tissue, thereby suppressing immune cell development and antibody production [3–8]. In addition, patients suffering from these diseases are often treated with cytotoxic chemotherapy which often leads to depletion or prolonged suppression of hematopoiesis with various degrees of lymphopenia and neutropenia [5, 9].

The development and use of monoclonal antibodies has improved the outcome of B-cell lymphomas [10]. Over the years many different antibodies against various B-cell or plasma cell targets like CD20, CD19, CD22 or CD38 have been developed. Especially the widespread use of rituximab, a B-cell directed monoclonal antibody against CD20 has improved progression-free and overall survival over the last years in B-cell lymphomas [11].

Depending on their targets, monoclonal antibodies deplete B-, T-, or plasma cells which can cause reduced immunoglobulin production and/or inhibited immune-cell-function [3, 12]. One of the most used and studied monoclonal antibodies is the anti-CD20 antibody rituximab which is able to induce a secondary immunodeficiency syndrome with hypogammaglobulinemia and resulting increased infection rate [13–15].

Over the years a continuous flow of new antitumor agents ranging from inhibitors of Bruton's tyrosine kinase affecting B-cell signaling to bispecific T-cell engager antibodies targeting CD19 have been developed [3, 16, 17]. These therapies are very effective in tumor treatment but can also cause severe side effects with increased incidences of immunodeficiency syndromes and infections [18].

As more and more patients are treated with these new therapies the number of patients with clinically significant immunodeficiency syndromes increases. Prophylactic infection management becomes therefore more and more important. In multiple myeloma and chronic lymphocytic leukemia (CLL), in which infections are a major cause of death, disease and therapy related immunodeficiencies have been studied for a long time [1, 2]. As a consequence, over the years immune surveillance and prophylactic treatment strategies were developed and are now widely used. Part of these strategies are regular patient contacts as well as the measurement of antibody and immune cell levels (T- and B-cell fractions) [19]. Guided by the obtained results different prophylactic strategies can be employed including vaccination and prophylactic antibiotic treatment. In the case of clinically significant secondary immunodeficiency which is defined by low immunoglobulin levels and an increased rate of infections, patients are usually treated with vaccination and prophylactic antibiotics as a first step measure [20, 21]. If these measures fail, regular immunoglobulin replacement is an effective therapy [22–24].

In patients with CLL and multiple myeloma it has been shown in various analyses that regular immunoglobulin substitutions significantly decrease the rate of infections [25–27]. As a result, prophylactic immunoglobulin substitutions have become a well established and widely used treatment strategy for hematological patients with secondary immunodeficiency which is also recommended by European guidelines (EMA/CHMP/BPWP/94038/2007 Rev. 5, Committee for Medicinal Products for Human Use). Of note, this kind of therapy can now be administered either intravenously or subcutaneously.

The available data of infection prevalence and severity in patients with hematological diseases were mostly generated in controlled trials whose studied population usually don’t represent the average population. This bias can be explained by variably strict study exclusion criteria and by focusing on patient populations that require treatment for their underlying hematological diseases. In addition many studies mostly include younger patients which may underestimate the risk of infections in older patients, if the obtained data are used to generalize the risk of infections [28, 29].

Another data resource to better understand the risk of infections in older patients with hematological diseases represent data from healthcare providers. However, these data are usually limited by the fact that patients not always consult their treating physician in cases of infections and therefore possibly underestimate the risk of infections. In addition, these data are usually not very detailed and only allow a broad analysis [30]. In conclusion, more data analyzing the risk of infections for patients with hematological diseases, especially for older patients, are necessary to better understand the risk in these patients.

In order to obtain a more comprehensive picture, we collected real life data from patients with indolent lymphomas and a matched control group (CG).

## Methods

### Study design

We conducted a monocenter longitudinal prospective study using a participant-reported telephone survey in which we collected real life data from patients with indolent lymphomas undergoing therapy or who were regularly monitored in a watch and wait approach in a German hematology/oncology outpatient center. Patients and the age and sex matched CG were regularly interviewed for the presence of infections, its treatment and if medical assistance was required. All relevant data regarding therapy and medical history were collected prospectively and analyzed statistically. Infections were evaluated every four weeks in the course of one year for patients and a CG. Patients' treatment data were assessed additionally. The design was hypothesis-generating.

### Patients

All patients who suffered from indolent B-non-Hodgkin lymphomas (B-NHLs) and received treatment or were monitored in an hematology/oncology group practice were eligible. All eligible patients who gave written informed consent were included.

### Setting and participants

All suitable patients were written to on behalf of the treating physicians. They were informed in detail about the project and had the opportunity to ask their doctors questions. Reminders were not sent nor were the patients contacted in another way. Written informed consent was obtained from the patients by sending the consent form to the center in a prepaid envelope.

Additionally, the patients' treatment data were linked to the interview data. Three subgroups were formed within the patient group: 'no immunodeficiency, no IgG substitution', 'non-symptomatic immunodeficiency, no IgG substitution' and 'symptomatic immunodeficiency, IgG substitution'.

Patients with IgG levels below 6 g/l or IgG subclass deficiency were categorized as immunodeficient. The cut-off was chosen as values below 6 g/l correlate with infection risk and poor functional antibody levels [31].

The diagnosis of IgG subclass deficiency was made by the treating physician. Immunodeficient patients were further categorized in symptomatic, if they had suffered from two infections that required antibiotic treatment in the previous year or asymptomatic if they had not. This categorization was used in order to have clear criteria that could be easily interrogated by phone.

Furthermore, a CG of healthy volunteers with a comparable distribution in terms of age and sex and not suffering from hematological or immunosuppressive diseases known to have effects on the immune system (e.g. diabetes mellitus, HIV infection / AIDS, cirrhosis of the liver) was questioned. This group was similarly contacted by telephone every four weeks over the course of one year (up to 12 times). The CG was recruited with the help of a commercial market research institute. CG participants were paid an incentive of 10 Euro per interview.

Patients and CG assessed their infections in the past four weeks retrospectively with the help of a short questionnaire. The survey was carried out between July 2017 and July 2018 and administered in German.

### Variables

The frequency of infections and of infections requiring antibiotics was our primary outcome. Secondary outcomes were duration and treatment of infections, hospitalizations and sick leaves. The questionnaire was short and non-validated (Additional file 1). It was comparable to a patient diary with the focus on infections. Age and sex as potential confounding variables were analyzed for differences.

Patients' comorbidities were assessed using the age-adapted Charlson comorbidity index (aaCCI) in order to predict the risk of death. The aaCCI is widely used and includes 19 health conditions that are associated to an increased mortality. Each comorbidity is based on its severity rated with 1–6 points, additional points (0–7) are added age-dependent.

### Sample size

544 patients were eligible due to their diagnosis. An assumed response rate of at least 30% would have resulted in about 160 valid interviews. From a pragmatic point of view we decided to conduct the study without a priori calculation of sample size and to contact all patients and accordingly about 150 participants in the CG. A post hoc power analysis with regard to the primary outcome has been done with the help of G*Power version 3.1.9.2 [32].

### Statistical methods

Data was analyzed with IBM SPSS Statistics, version 19. Frequencies, percentages, medians, means and standard deviations were calculated to describe the data. Data were analyzed for patients and CG as well as subgroups. All tests of statistical significance were two-sided, statistical significance was defined as a *p* value of 0.05 or less. The Bonferroni method was applied to correct for multiple testing, resulting in *p*-values of 0.00625. Statistical significance was checked using 4 t-tests for independent samples and 4 one-factor analyses of variance (ANOVA) with pairwise treatments of missing values and, if applicable, Games-Howell post hoc tests. Homogeneity of variances was checked with the Levene test, normal distribution with the Shapiro–Wilk test. Non-parametric tests were not used. In case of missing prerequisites, the Welch test was used in the ANOVA. In the t-tests no test for normal distribution was performed because the comparison groups were sufficiently large (n > 30). Outliers, on the other hand, were checked.

Descriptive statistics included demographics, treatment data and the results of the questionnaires. No participant was excluded from the analysis due to incomplete data. Because of the better comparability with existing studies, the number of infections, the number of days taking antibiotics and the number of days in hospital due to infections was standardized for 1000 days.

### Data protection and ethical approval

Data were captured pseudonymized. Written informed consent was obtained before the first interview from the patients, the CG verbally agreed to be interviewed by telephone The study had been approved by the ethics committee of Rhineland-Palatinate, Germany (837.498.16 (10816)).

## Results

From July 2017 to July 2018 227 patients with B-NHLs as well as 145 individuals of a matched CG were contacted monthly and interviewed for the presence of infections.

161 patients (70.9%) and 113 healthy volunteers (77.9%) completed the entire 12-month survey. Median in both groups was 12 interviews, with ranges from 2–12 (patients) and 7–12 (CG) interviews. The mean number of interviews was 11.36 (patients) and 11.10 (CG) respectively.

A post hoc power analysis with regard to the primary outcome mean number of infections per 1000 days revealed a statistical power (1 − β) of 0.98 under the following assumptions: two tailed t-test, calculated effect size d = 0.52, adjusted α error probability of 0.00625 and sample sizes of n = 227 and n = 145.

The mean age of patients with lymphoma was 68.8 years (standard deviation (SD): 9.01 years) and 67.2 years for the CG (SD: 11.42 years) (*p* = 0.164). Both groups were well balanced for sex and age (Table 1).

**Table 1:** Demographics

		Patients		Control group	
		Number	Mean	Number	Mean
Age		n = 227	68.8 years	n = 145	67.2 years

		Number	%	Number	%
Age groups	69 years or younger	n = 118	52.0	n = 74	51.0
	70 years or older	n = 109	48.0	n = 71	49.0
Sex	Female	n = 81	35.7	n = 51	35.2
	Male	n = 146	64.3	n = 94	64.8
Hematological diagnosis	CLL	n = 76	33.5		
	Follicular lymphoma	n = 50	22.0		
	Multiple myeloma	n = 50	22.0		
	Waldenstrom's disease	n = 20	8.8		
	Hairy cell leukemia	n = 16	7.0		
	Mantle cell lymphoma	n = 5	2.2		
	Marginal zone lymphoma	n = 5	2.2		
	Lymphoplasmocytic lymphoma	n = 3	1.3		
	Small cell B-cell lymphoma	n = 1	0.4		
	Multiple myeloma and CLL	n = 1	0.4		
Systemic therapy	No systemic therapy	n = 50	22.0		
	No systemic therapy during observation	n = 116	51.1		
	Systemic therapy during observation	n = 61	26.9		
IgG replacement	no IgG Replacement	n = 171	75.3		
	no IgG Replacement during observation	n = 14	6.2		
	IgG replacement during observation	n = 42	18.5		
Patient groups	No immunodeficiency No IgG replacement	n = 105	46.3		
	Non-symptomatic immunodeficiency No IgG replacement	n = 60	26.4		
	Symptomatic immunodeficiency IgG replacement	n = 51	22.5		
	Symptomatic immunodeficiency No IgG replacement	n = 6	2.6		
	No or non-symptomatic immunodeficiency. IgG replacement	n = 5	2.2		
Death during observation	Deceased	n = 4	1.8		
	Not deceased	n = 223	98.2		
Cause of death	Comorbidity	n = 2	50.0		
	Lymphoma	n = 1	25.0		
	other	n = 1	25.0		

Patients in the lymphoma group suffered from various B-cell non-Hodgkin lymphomas (B-NHLs). CLL was the most common B-NHL representing 33.5% (76 patients) followed by follicular lymphoma (22.0%, 50 patients), multiple myeloma (22.0%, 50 patients), Waldenström macroglobulinemia (8.8%, 20 patients) and hairy cell leukemia (7.0%, 16 patients). The patient cohort also included rare B-NHL subtypes. For detailed information see Table 1. 177 patients (78.0%) received treatment during observation or had received therapy before observation. 50 patients (22.0%) never have had treatment. Different treatment consisted of cytotoxic chemotherapy in 45.8% (n = 81), chemotherapy + anti-CD20 therapy in 57.1% (n = 101), anti-CD20 therapy in 31.1% (n = 55). 18.1% (n = 32) had immunomodulatory agents, 13.6% antibodies (no anti-CD20) ± chemotherapy (n = 24) and 12.4% (n = 22) radiation. Based on the measured IgG blood levels and clinical symptoms, patients were categorized as immunodeficient with the subgroups of symptomatic and asymptomatic patients. 46.3% of patients (n = 105) were categorized as patients without immunodeficiency.

Most symptomatic patients received regular IgG substitution. In detail, 25.1% of patients (n = 57) had a symptomatic IgG deficiency and 89.6% of these received regular therapeutic immunoglobulin substitutions either before (n = 9) or during (n = 42) the time of observation (n = 51). Interestingly, about 2.6% of all patients (n = 6) didn’t receive regular immunoglobulin substitutions although categorized as symptomatic. 60 of 227 lymphoma patients (26.4%) were categorized as immunodeficient due to decreased immunoglobulin blood levels but received no specific treatment as they had no increased infection rate. 5 patients (2.2%) received IgG replacement although they showed no (symptomatic) immunodeficiency according to our definition. For detailed patient information see Table 1.

**Fig. 1:**
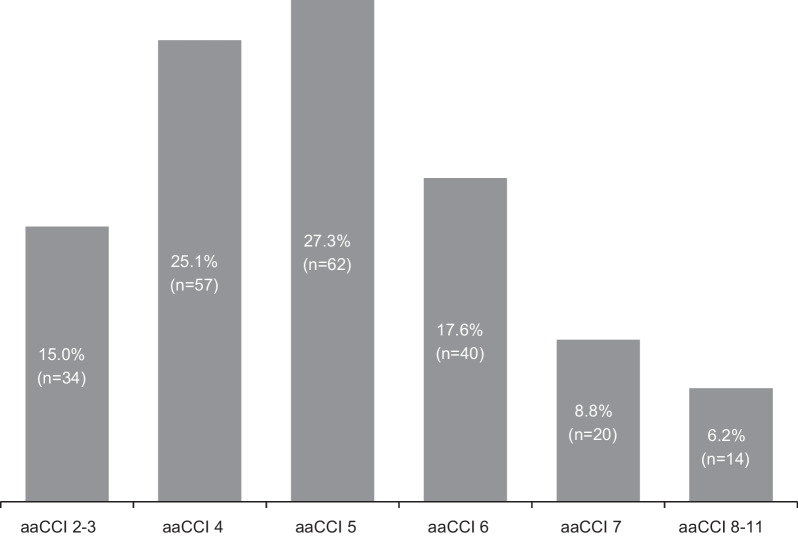
Age adjusted Charlson Comorbidity Index—aaCCI at the beginning of the observation

Figure 1 shows the distribution of the aaCCI of all 227 patients at the beginning of the observation period.

### Number of infections

Patients suffering from B-NHLs had statistically significant more infections per 1000 days compared to the CG (*p* < 0.001). Patients had 11.66 infections in mean, SD was 9.93. The mean number in the CG was 7.13 infections per 1000 days (SD: 7.15). A t-test was conducted to check for statistical significance. 2 outliers were found with 48 and 71 infections per 1000 days which seemed to be possible and these data were not excluded.

**Fig. 2:**
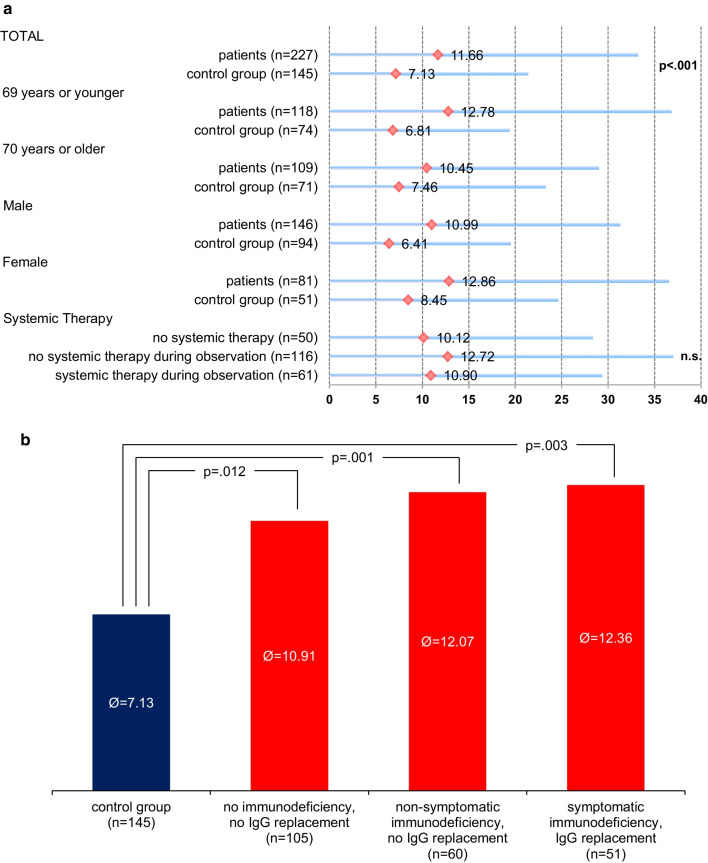
Mean infection numbers per 1000 days

This increased infection rate was a consistent finding and could be shown for male and females as well as for different age groups. Figure 2a, b depict the mean infection numbers for subgroups. Infections were mostly respiratory infections (46.7%) followed by skin (11.9%) and urinary infections (4.7%). 47.0% were not further specified.

As expected patients suffering from immunodeficiency defined by decreased IgG levels had more infections per month than patients without documented immunodeficiency. Means and standard deviations were as follows: CG 7.13 (SD: 7.15), patients without immunodeficiency 10.91 (SD: 10.90), patients with non-symptomatic immunodeficiency 12.07 (SD: 8.33) and symptomatic patients 12.36 (SD: 9.47). We conducted a one-way ANOVA to assess the number of infections per 1000 days in the three patient subgroups and the CG. There were two outliers, according to inspection with a box-plot, which were not excluded from the analysis. Data was not normally distributed (Shapiro–Wilk test, *p* < 0.001) and homogeneity of variance was not given (Levene's test, *p* = 0.011). The number of infections differed statistically significant for the CG and the subgroups according to the applied Welch test: Welch’s F(3, 140.53) = 9.093, *p* < 0.001, η^2^ = 0.060. The patient subgroups did not differ significantly. Games-Howell post-hoc tests revealed a significant difference between CG and patients without immunodeficiency (*p* = 0.012, MDiff = 3.78, 95%-CI[0.62, 6.94]), patients with non-symptomatic immunodeficiency (*p* = 0.001, MDiff = 4.94, 95%-CI[1.73, 8.15]), and patients with symptomatic immunodeficiency (*p* = 0.003, MDiff = 5.23, 95%-CI[1.40, 9.05]).

However, although the subgroup of patients without immunodeficiency suffered from less infections than patients with immunodeficiency, its infection rate was still much higher than the infection rate of the CG. This finding points out that B-NHL patients have an increased risk of infections even when IgG levels are normal.

Patients with symptomatic or asymptomatic immunodeficiency had comparable infection rates suggesting a therapeutic effect of regular immunoglobulin substitutions. Of note, patients categorized as asymptomatic and therefore not treated with regular immunoglobulin substitutions, suffered from an increased infection rate questioning their categorization as asymptomatic.

Analysis of infections per 1000 days according to treatment revealed the following results: Patients without therapy had 10.12 (SD: 8.13) infections in mean, patients who had no therapy in the observation period had 12.72 (SD: 11.54) infections and patients who received systemic therapy in the observation period had 10.90 (SD: 7.58) infections per 1000 days. These numbers did not differ statistically significant according to the applied Welch test: Welch’s F(2, 129.34) = 1.476, *p* = 0.232.

### Antibiotic treatment

**Fig. 3:**
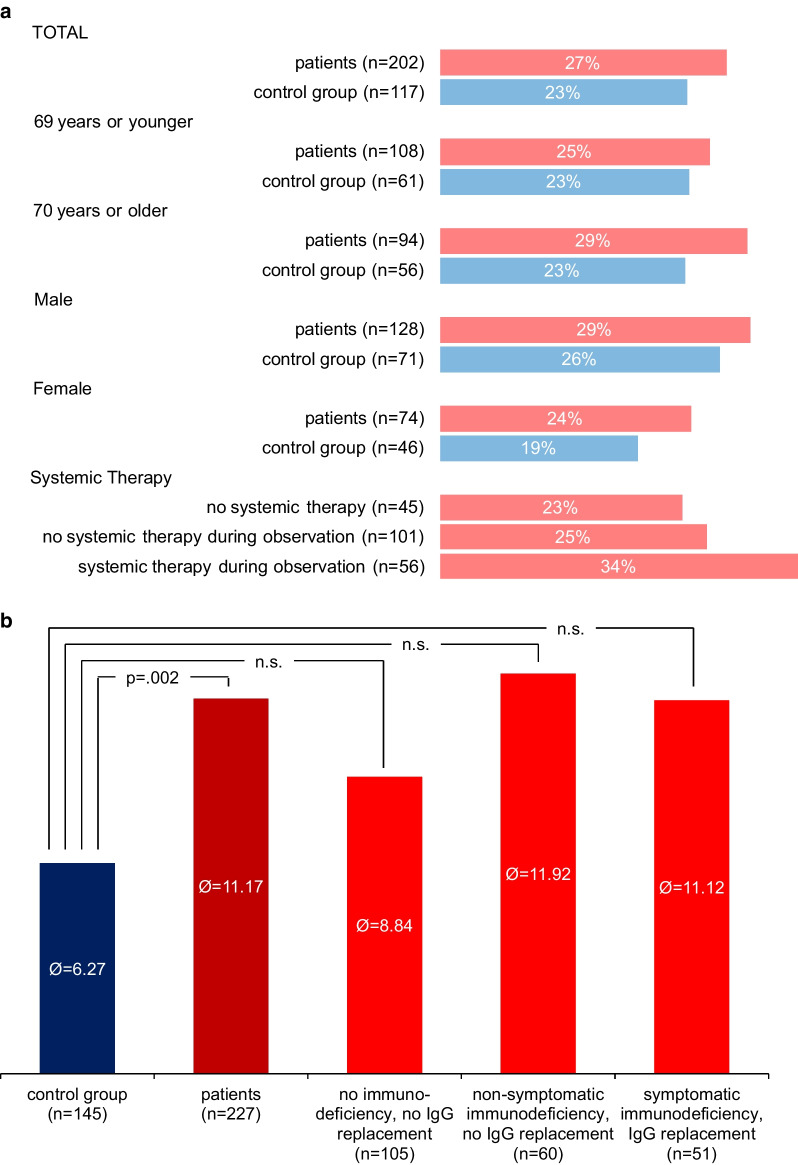
**a** Frequency of antibiotic treatment in infections. **b** Duration of antibiotic treatment per 1000 days

Figure 3a depicts the frequency of antibiotic treatment in participants suffering from infections.

A higher rate of antibiotic treatment was observed in patients compared to the CG. Antibiotics intake in 1000 days was analyzed using '0' days in participants who had had no infections. Patients took in mean 11.17 (SD: 19.05) days antibiotics, the mean value in the CG was 6.27 (SD: 11.55) days. Two outliers with 77 and 197 were observed, data were not excluded. The applied t-test was statistically significant (*p* = 0.002).

The mean duration of antibiotics intake was as follows in the patient subgroups: no immunodeficiency 8.84 (SD: 15.23) days, non-symptomatic immunodeficiency 11.92 (SD: 15.39) days and symptomatic patients 11.12 (SD: 12.47) days. We conducted a one-way ANOVA to assess the number of days with antibiotics intake per 1000 days in the three patient subgroups and the CG. Data of two outliers, according to inspection with a box-plot, were not excluded. Data was not normally distributed (Shapiro–Wilk test, *p* < 0.001) and homogeneity of variance was not given (Levene’s test, *p* = 0.004). The applied Welch test (Welch’s F(3, 142.71) = 3.479, *p* = 0.018) was considered statistically not significant due to the to multiple testing adapted *p*-value of *p* = 0.00625.

Although statistically not significant, patients with documented immunodeficiency (symptomatic or asymptomatic) required more antibiotic treatment than B-NHL patients without immunodeficiency. However, although no immunodeficiency was detectable these patients still required more antibiotic treatment than the CG. Figure 3b depicts the mean duration of antibiotic treatment per 1000 days for all participants.

All analyzed lymphoma subgroups required significantly longer antibiotic treatment in comparison to the CG.

### Hospitalization

**Fig. 4:**
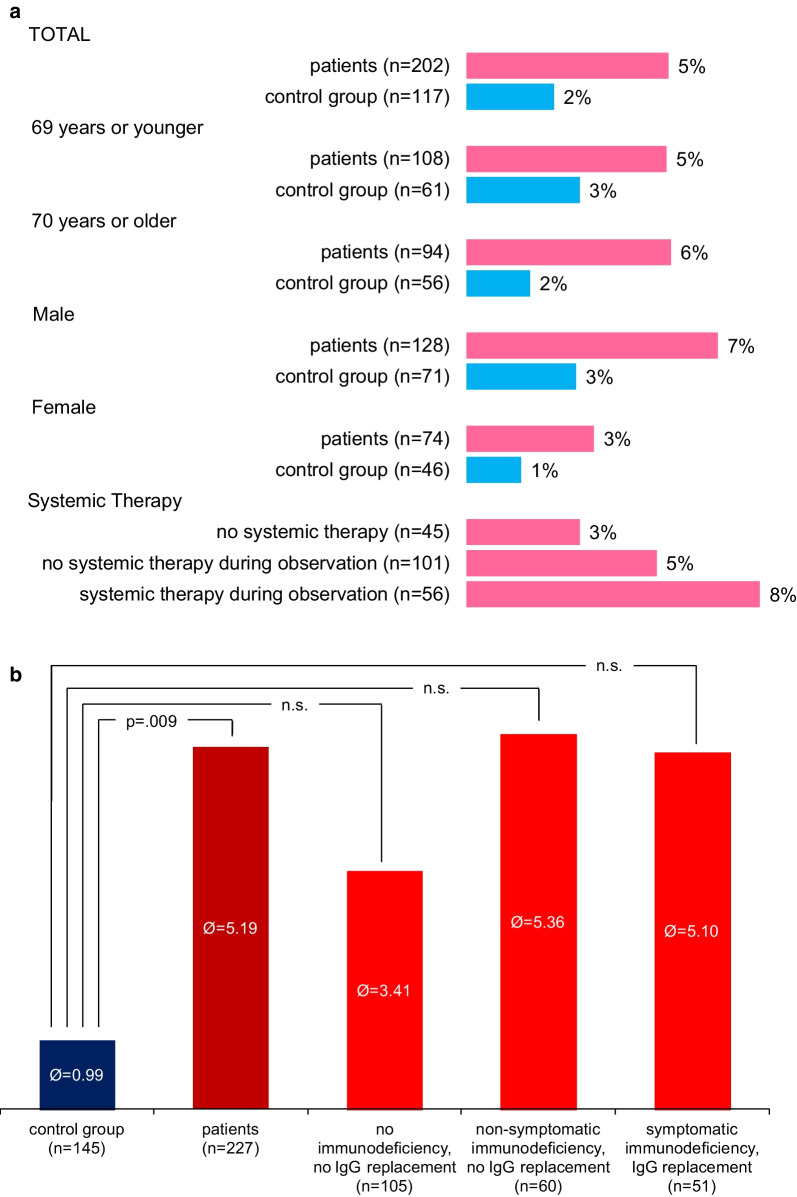
**a** Frequency of hospitalizations due to infections. **b** Duration of hospital stays in days per 1000 days

As hospitalization is a good marker for severe infections we also compared days in hospital / 1000 days for B-NHL patients and our CG. As expected and in line with the previous findings we also found a much higher hospitalization rate for B-NHL patients than in the matched CG. This effect was again detectable in all subgroups. Figure 4a depicts the frequency of hospitalizations due to infections in participants suffering from infections.

All patient subgroups had a higher rate of hospitalization compared to CG during the time of observation.

Duration of hospitalizations due to infections in 1000 days was analyzed using '0' days in participants who had had no hospitalization due to an infection. Due to this methodological procedure many outliers could be observed. Data were not excluded, because the values seemed to be possible. Patients stayed in mean 5.19 (SD: 22.26) days per 1000 days in hospital, the mean value for the CG was 0.99 (SD: 7.34) days (*p* = 0.009).

As shown before patients with detectable immunodeficiency had the highest risk of infections irrespective of the classification as symptomatic or asymptomatic. Mean values in days per 1000 days were as follows: patients without immunodeficiency 3.41 (SD: 18.07) days, patients with non-symptomatic immunodeficiency 5.36 (SD: 18.15) days and symptomatic patients 5.10 (SD: 12.19) days.

Figure 4b depicts the mean duration of hospitalizations in days per 1000 days for all participants.

All analyzed lymphoma patient subgroups required longer in-patient treatment in comparison to the CG. The conducted one-way ANOVA assessed the number of days in hospital due to an infection per 1000 days in the three patient subgroups and the CG. There were many outliers, which were not excluded. Data was not normally distributed (Shapiro–Wilk test, *p* < 0.001) and homogeneity of variance was not given (Levene’s test, *p* < 0.001). The number of days in hospital did not differ statistically significant for the CG and the patient subgroups: Welch’s F(3, 125.31) = 2.851, *p* = 0.040.

## Discussion

In our prospective study we can show an increased infection rate of patients suffering from B-NHLs in comparison to an age and sex matched CG.

Patients were assessed based on their measurable immunoglobulin levels. Immunodeficient patients were further categorized in symptomatic, if they had suffered from two infections that required antibiotic treatment in the previous year. The remaining immunodeficient patients were categorized as asymptomatic.

Overall, patients with B-NHLs suffered from more infections compared to the CG. As expected for B-NHL patients, those with immunodeficiency had an even higher risk of infections. Interestingly, patients with symptomatic immunodeficiency and IgG replacement had comparable numbers of infections to patients with asymptomatic immunodeficiency. The most plausible explanation for this observation is that regular IgG infusions have a strong infection preventing effect suggesting that otherwise the rate of infections for this subgroup would be much higher. However, even regular IgG infusions cannot lower the risk of infections to the level of the CG which underlines the fact, that pathophysiological effects other than decreased immunoglobulin levels cause the observed clinical significant immunodeficiency.

Our data demonstrate that although scored asymptomatic, patients with measurable immunodeficiency do have an increased risk of infections. This also shows that the clinical assessment which decides about the treatment with regular immunoglobulin substitutions does miss patients that are at increased risk of infections. These patients can be easily identified by routine laboratory parameters like the measurement of IgG levels. With this simple analysis it would be possible to identify more patients with increased risk of infections. It also raises the question if patients with immunodeficiency should be generally treated with regular immunoglobulin substitutions, independent from the clinical assessment. Our data support this but there are still data missing and important points to consider. Over the last years the outcome of lymphoma patients has improved significantly by the development of new anti-tumor drugs [33]. As a result patients live longer and more patients develop immunodeficiencies that require treatment. Regular immunoglobulin replacement is an expensive therapy regimen with considerably long infusion times that requires specialized outpatient clinics with well trained medical staff. Although in recent years subcutaneous immunoglobulin substitutions are more and more used, the therapy regimens remain expensive which is an important argument to restrict this therapy to symptomatic patients [34].

However, the more widespread use of regular immunoglobulin substitutions may reduce the overall infection rate of lymphoma patients which may lead to less antibiotic treatment and especially less hospitalization which would thereby prevent expensive therapies. As our study was performed before the outbreak of the COVID19 pandemic, we cannot draw any conclusions on the effect immunoglobulin infusuions may have had on the incidence of severe COVID 19 cases. Future studies are needed to show if there is protection from such a strategy, especially as CD20 treated lymphoma patient are at high risk for complications with fatal infections. [35].

The main bias of our study is that it relies on patient self-assessments to analyze the number and treatment of infections. However, there are also advantages to patient self assessment as it probably provides a more complete picture of patient infections. In cases of minor infections patients may have contacted only their GP or not consulted a physician at all. In these cases infections would have never been documented. Since we wanted to capture all infections, patients’ assessments were in our opinion the only possible data source.

So far the existing data are not sufficient to make a general recommendation to treat all indolent B-NHL patients with regular immunoglobulin substitutions. It is therefore necessary to conduct larger prospective trials that should also analyze the effects on survival.

## Conclusion

In conclusion we propose regular immune monitoring of all indolent lymphoma patients including the measurement of immunoglobulin levels and regular appointments for clinical assessment. With this approach patients at increased risk of infections can be identified early and lined up for prophylactic treatment.

## Supplementary Information

**Additional file 1**. Questionnaire.

## Data Availability

The datasets used and analyzed during the current study are available from the corresponding author on reasonable request.

## Abbreviations

aaCCI: Age-adapted Charlson comorbidity index
ANOVA: Analyses of variance
B-NHLs: B-cell non-Hodgkin lymphomas
CG: Control group
CLL: Chronic lymphocytic leukemia
