# Supplementary material for: Patients with indolent lymphomas are at high risk of infections: experience from a German outpatient clinic
Source: BMC Immunol. 2023 Jan 11;24:2. doi: 10.1186/s12865-022-00536-x (PMC9833869; doi:10.1186/s12865-022-00536-x)
Supplement: Supplementary file 1 — Additional file 1. Questionnaire. [file 12865_2022_536_MOESM1_ESM.docx]

**Questionnaire**

1. Have you had one or more infections in the **last 4 weeks**? By this we mean any form of inflammation, infection or contagion?

- yes *⇨ question 2*
- no *⇨ end*
- not sure / do not know *⇨ end*

1. How many **different** infections have you had in the **last 4 weeks**?

- ___________ infections

Let's talk about all these infections in detail!
*[repeat the following questions 3-5 for the mentioned number of infections!]*

1. Has this 1^st^ / 2^nd^ / 3^rd^ / x^th^ infection newly appeared in the last 4 weeks or did you have this infection before?

- this infection has newly appeared in the last 4 weeks
- this infection has **not** newly appeared in the last 4 weeks, it already existed before
- not sure / do not know

1. And how long do you have or have you had this 1^st^ / 2^nd^ / 3^rd^ / x^th^ infection approximately?

- less than 1 week
- 1-2 weeks
- 2-4 weeks
- longer than 4 weeks
- not sure / do not know

1. What kind of infection did you have? Where was this infection located?
   *[if necessary, provide alternative answers!]*

- throat
- bronchial tubes/lungs
- urinary tract
- skin
- others
- not exactly localisable / several localisations
- not sure / do not know

1. Have you seen a doctor, whether a general practitioner or a specialist, for (one of) these infection(s)?

- yes
- no
- not sure / do not know

1. Have you taken antibiotics in the **last 4 weeks** due to an infection / infections?

- yes, due to an infection *⇨ question 8*
- I take antibiotics prophylactically, but not because of the current infection(s)
  *⇨ question 10*
- no *⇨ question 10*
- not sure / do not know *⇨ question 10*

1. What was the name of the antibiotics?

- ______________________________________________________

1. And how many days did you take the antibiotics in total?

- still taking antibiotics (s)
- _______ days

1. Have you taken any other medicines for your infection(s) in the **last 4 weeks**? We mean, for example, drugs against viruses or fungi? *[multiple responses possible!]*

- yes, antivirals
- yes, antifungals
- yes, steroids
- yes, non-steroidal anti-inflammatory drugs (NSAIDs)
- yes, but exact drug not known
- no
- not sure / do not know

1. Were you on sick leave due to your infection(s)??

- yes *⇨ question 12*
- no *⇨ question 13*
- not applicable, as pensioner / retiree / job seeker or similar *⇨ question 13*

1. How many days were / are you on sick leave in total?

- _______ days

1. Were you hospitalized due to your infection(s)?

- yes *⇨ question 14*
- no *⇨ end*

1. How many days **in total** were you hospitalized due to your infection(s)?

- _______ days

Thank you very much, those were all my questions!

We will get back to you in about 4 weeks and ask you some quick questions again.
